# Chemical Characterization and In Vitro Gas Production Kinetics of Alternative Feed Resources for Small Ruminants in the Maltese Islands

**DOI:** 10.3390/metabo13060762

**Published:** 2023-06-17

**Authors:** Grazia Pastorelli, Kalliroi Simeonidis, Massimo Faustini, Angelo Le Mura, Mariagrazia Cavalleri, Valentina Serra, Everaldo Attard

**Affiliations:** 1 Department of Veterinary Medicine and Animal Sciences, University of Milano, Via dell’Università 6, 26900 Lodi, Italy; kalliroi.simeonidis@studenti.unimi.it (K.S.); cavalleri@fbn-dummerstorf.de (M.C.); valentina.serra@unimi.it (V.S.); 2 Indipendent Researcher, 28053 Castelletto sopra Ticino, Italy; angelolemura@libero.it; 3 Research-Institute for Farm Animal Biology (FBN), Institute of Nutritional Physiology, Wilhelm-Stahl-Allee 2, 18196 Dummerstorf, Germany; 4 Division of Rural Sciences and Food Systems, Institute of Earth Systems, University of Malta, MSD 2080 Msida, Malta; everaldo.attard@um.edu.mt

**Keywords:** gas production technique, alternative feed, small ruminants, chemical analyses, antioxidant activity, polyphenols

## Abstract

The ever-increasing human population, the problem associated with climate change and recent crises—COVID-19 disease and trade conflicts—all impacted on the availability and cost of animal feed raw materials. This is clearly visible in realities which heavily rely on importation such as islands and small states, where producers involved in the agricultural sector were strongly affected by the sharp increase in prices. To deal with these global issues, alternative resources are perceived to replace conventional ingredients. This work aimed at assessing the nutritive value of different resources (sheep feed, mature carob, Maltese bread, wild asparagus, prickly lettuce, and loquat) for small ruminants present in the Maltese Islands, analyzing their chemical composition, gas production kinetics and antioxidant properties. In general, the variation in chemical composition resulted in different rumen fermentation kinetics (*p* < 0.007). The ratio between GP-24 h and GP-48 h was higher in Maltese bread than other substrates; loquat, prickly lettuce and wild asparagus showed lower fermentation kinetics in accordance with their high NDF and ADF contents. The antioxidant activity may be partially related to the polyphenolic content that was higher in wild asparagus, prickly lettuce and loquat. All feed characteristic confirmed their potential to be included as ingredients in ruminant diets and as a source of fiber.

## 1. Introduction

As a result of rising consumption, the world’s natural resources are coming under increasing pressure from the ever-growing human population, which is projected to reach 10 billion by 2050 [1]. Additional factors to this scenario include the problems associated with climate change and recent crises, COVID-19 disease and trade conflicts, exacerbated existing food systems’ vulnerabilities. Particularly, the global feed industry was faced with a situation that was never faced before: several external factors such as dangerous weather conditions in the growing season, as well as geopolitical issues, the impact of reliance on imports for staple foods, fodder for livestock, fertilizer and fuel impacted the availability and cost of feed raw material, which directly affected animal production. The impact is clearly visible in realities that heavily rely on importation, such as in the case of the Maltese Islands and other islands and small states which remain highly vulnerable to food price and foreign exchange volatility. To overcome the problems associated with the increase in the raw material prices, many farmers started to resort to alternative ingredients and new, potentially local sources to help meet low-cost formulation objectives.

Despite the small size of the Maltese Islands, they host a wide number of plants. Plant biodiversity, with its 1264 vascular species, is mainly attributed to the strategic position of Malta within the Mediterranean [2]. Some of these plants, present locally and in abundance, were considered as a potential alternative source for animal nutrition in order to mitigate the production costs of products of animal origin. Using natural vegetable resources may be an inexpensive, feasible and effective means to reduce production costs and will likely improve the quality of the products [3].

Although, in recent years, the interest in the use of alternative feed sources grew considerably, many of these were noy yet sufficiently investigated for their characteristics and properties. Therefore, there is a need for more information on the chemical composition and nutritive value of these resources to expand knowledge on their potential use as feedstuffs before making recommendations about their inclusion in animal diets. Chemical composition, in vitro digestibility and in vitro rumen fermentation kinetics are useful tools for feed evaluation, especially for ruminants. The in vitro techniques represent biological models that simulate the in vivo digestion processes with different levels of complexity. Compared to in vivo experiments, in vitro methods have the advantage not only of being less expensive, less time-consuming and more humane but allow for a more precise control of experimental conditions and for screening of a large number of materials in a relatively short time. The gas production test is an in vitro valuable tool in the evaluation and selection of feeds for ruminants, since it provides information on the fermentability, digestibility and nutritional value of feeds. Efficient fermentation implies a greater use of feed nutrients by rumen microorganisms, which translates into a higher conversion efficiency of feed into final products, such as volatile fatty acids and microbial protein. Thus, this contributes to a better diet formulation and an improvement in the feeding efficiency of ruminants. The aim of this study was to assess the nutritive value of alternative feeds resources for small ruminants in the Maltese Islands, analyzing their chemical composition, gas production kinetics and antioxidant properties.

## 2. Materials and Methods

### 2.1. Feed Resources

Five raw materials, Maltese bread, wild asparagus, loquat, prickly lettuce, mature carob and a commercial sheep feed, were used as substrates. Representative samples of each substrate, except for the sheep feed and Maltese bread, were collected from the rural area of the Maltese Islands. Maltese bread is a crusty sourdough bread, typical of the Maltese Islands. The parts of the feed sources tested are illustrated in Table 1. The sheep feed was composed of semolina, bran, corn, soya, alfalfa, sugarcane pulp, calcium carbonate, whereas the Maltese bread was made up of wheat flour, yeast, salt and water.

#### Analysis

All the samples were oven dried at 40 °C for 48 h and were then ground to pass a 1 mm sieve and stored for subsequent analysis prior to NIR analysis.

### 2.2. Proximate Analysis

Near infrared spectra of feed samples were acquired using a SpectraStar NIR spectrophotometer (the Unity^®^ Scientific SpectraStarTM XT NIR Analyzer Series). Around 30 g of a bulk of feed samples were transferred to the quartz sample holder, sealed with a gold reflector, and placed over the sample window (1 cm of diameter), to ensure direct contact and minimize noises due to light scattering. Spectral data were acquired and recorded as absorbance spectra in wavelength range from 1400 to 2500 nm. During spectral acquisition, the sample was set to rotate and read three times (with a total of ninety determinations per sample) to ensure uniformity. All proximate analyses were conducted in triplicate. Parameters included dry matter (DM), crude protein (CP), ether extract (EE), neutral detergent fiber (NDF), acid detergent fiber (ADF) and total ash.

### 2.3. Inoculum Sources

The rumen fluid was collected from slaughtered sheep. In Malta, sheep breeds include the Comisana, the East Friesian, the Maltese and the Crossbreed. Rumen fluid was collected at a slaughterhouse from culled sheep previously fed under controlled conditions (i.e., commercial sheep feed and, additionally, wheat, sulla and clover), slaughtered in good health and transported from farms located near the slaughterhouse. The samples were collected from three sheep, which were (1 L per sheep) mixed and then treated with DMSO (5%) as cryoprotectant and delivered to laboratory within 30 min from slaughter. For the frozen preservation conditions, the tubes with 4 mL inocula containing a dimethyl sulfoxide (DMSO)/saline mixture were placed in a freezer at −80 °C to maintain inoculum homogeneity throughout the experiment.

### 2.4. In Vitro Gas Production

In vitro fermentation of the samples was conducted in an automated ANKOM gas production system (ANKOM RF; Ankom Technology Corp., New York, NY, USA). Briefly, buffered solution [4] was prepared and placed in a water-bath at 39 °C under continuous flushing with CO_2_. Then, five jar bottles (250 mL) were filled with 78 mL of buffer solution along with 0.5 g of randomly assigned feedstuff (two of each feedstuff and one controls) per run. Four milliliters of inoculum were transferred to each fermentation bottle that, in turn, were immediately attached to the Ankom system. Blanks contained only the medium, inoculum and sheep feed. Bottles were incubated at 39 °C for 72 h. The pH of the contents of these bottles was recorded (pH meter, Thermo Scientific Orion 4-Star) at time 0 and at the end of the experimental period, that is, 72 h. The pH of the medium and CO_2_ saturation at time 0 was controlled by a color change for the resazurin indicator from purple to pink/colorless. Pressure readings inside the bottles were recorded remotely every 10 min, through a wireless data logger system and the Ankom software (Gas Pressure Monitor, Ankom Technology Corp., Macedon, NY, USA) via a computer.

### 2.5. Physicochemical and Antioxidant Activity of Substrates Extracts

Prior to the assay, approximately 1 g of each dried subsample was allowed to macerate with 10 mL methanol in 50 mL centrifuge tubes. The supernatant was recovered and stored in the dark at −20 °C until analysis.

### 2.6. UV Analysis and Absorbance

The extracts (100 μL) were mixed with 900 μL of acidified methanol (1 M HCl:MeOH, 5:95) and scanned over a wavelength range of 200−800 nm in a UV-VIS spectrophotometer (Lightwave II, WPA). The following parameters were calculated:

Tint Ratio = (A_420_/A_520_) Color intensity = (A_420_ × DF) + (A_520_ × DF) + (A_620_ × DF)
Flavonoid Ratio = A_420_/A_520_
Anthocyanin content (mg/kg) = 1000 × V_s_ × DF × A_520_/ε

where A_280_, A_420_, A_520_, and A_620_ are the absorbance values at 280, 420, 520 and 620 nm, respectively; DF is the dilution factor (sample to diluent = 100 μL:900 μL = 10); V_s_ is the volume of extract per gram of plant material; and ε is extinction coefficient [58.3 mL/(mg·cm)]. Results are the mean values (LSmean ± SEM) of three replicates.

### 2.7. Folin–Ciocalteu Test for Polyphenols/Total Phenolic Content

The total phenolic content (TPC) in methanol extracts was estimated using Folin–Ciocalteu (FC) reagent according to [5]. Gallic acid standard solution (960 μg/mL) was serially diluted 1:2 up to 60 μg/mL to construct the calibration curve. The FC reagent was diluted 1:10 with deionized water, while sodium carbonate was prepared as a 1 M solution. Briefly, 10 μL of extracts were pipetted in triplicate in wells of a 96-well microtiter plate (Nunc^TM^ MicroWell^TM^, ThermoFisher Scientific^TM^,Waltham, MA, USA^,^). After adding 100 μL of FC reagent and 80 μL of sodium carbonate to each well, the plate was allowed to incubate at room temperature in the dark for 20 min and read at 750 nm on a spectrophotometer (SpectraMAX 340PC, Molecular Devices Corporation, San Jose, CA, USA). TPC was expressed as mg Gallic Acid Equivalents (GAE)/g. Results are the mean values (LSmean ± SEM) of three replicates.

### 2.8. Antioxidant Scavenging Assay

The DPPH free radical scavenging method is an antioxidant assay based on electron transfer which allow to calculate in a quick and simple way antioxidants by means of spectrophotometry. This assay was used as complementary method to evaluate the potential antioxidant activity by screening the DPPH free radical scavenging. The antioxidant activity of the substrates was determined using the microplate method [6].

Briefly, 100 μL aliquots of samples were transferred to wells in duplicates and in two sets. Subsequent one in two dilutions were carried out for these aliquots down the microtiter plate. One set was then treated with 100 μL methanol per well while the other set was treated with 100 μL DPPH solution per well. The plate was allowed to incubate at room temperature in the dark for 30 min and read at 517 nm in a microplate reader. Results were expressed as IC_50_ values. The values of IC_50_ denote the concentration of the sample that is required to scavenge 50% of DPPH free radicals. Results are the mean values (LSmean ± SEM) of three replicates.

### 2.9. Statistics Analysis

One-way ANOVA was performed with SPSS software (version 26.0 for Windows, SPSS Inc., Chicago, IL, USA). Duncan’s test was applied to assess significant differences among the variables (*p* < 0.05) in terms of FC, DPPH and anthocyanins parameters. Principal component analysis was conducted on all feed for all physicochemical parameters studied, using XLSTAT (Microsoft, version 19.4.46756, SAS Institute Inc., Marlow, Buckinghamshire, UK) software. The gas produced during the fermentation process results in an increase in pressure measured over the chosen interval and was converted into gas volume using the ideal gas law. The data for cumulative GP (as mL of gas produced per g dry matter (DM) with time) for each profile were processed by the three-parameters Gompertz sigmoidal function. The choice of such function was decided by using the Akaike Information Criterion (AIC), an estimator of prediction error. The Gompertz function (three parameters) is defined as:

$$y\left(t\right)=a\;*\;e^{-b\;*\;e^{-c\;*\;t}}$$

where:

*y*(*t*) is for the quantity of the gas produced at time t;

*a* = asymptotic value: estimated cumulative gas production;

*b* = latency time period;

*c* = growth rate;

*e* = Eulerian constant (*e* = 2.71828...). For each curve, the determination coefficient (R^2^) was calculated.

For cumulative, latency and rate analysis of variance (ANOVA) model, considering substrate as fixed factor was applied. Multivariate hierarchical cluster analysis was performed using the chemical composition, and gas production kinetics data was analyzed to study emerging groupings of the feed. The method used for hierarchical agglomerative classification was complete linkage clustering based on a furthest neighbor criterion, with the furthest pair of observations between groups used to determine (dis)similarity of the groups. The similarity and dissimilarity measures were calculated as squared Euclidean distances.

## 3. Results

### 3.1. Proximate Analysis

The proximate analysis of feedstuffs is shown in Table 2. The chemical composition was quite variable between sources. DM varied in a ranging from 93.47 (wild asparagus) to 87.75 g/kg DM (mature carob). CP varied in a ranging from 14.20 (sheep feed) to 5.70 g/kg DM (loquat). EE content was also quite variable, with values from 6.43 (loquat) to 0.01 g/kg DM (mature carob). In the same way NDF content varied, from 56.22 (mature carob) to 36.80 g/kg DM (loquat). A large variation among sources was observed in the ADF content, ranging from 46.38 (wild asparagus) to 4.36 g/kg DM (Maltese bread). Additionally, ash widely varied with valued from 17.08 (loquat) to 5.27 g/kg DM (mature carob).

### 3.2. In Vitro Gas Production and Fermentation Kinetics

**Figure 1:**
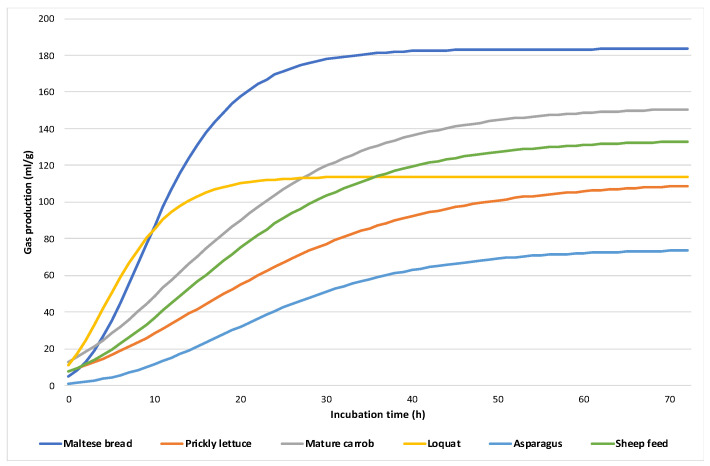
In vitro gas production (gas mL/g) of the tested substrates over time (h).

Results from in vitro gas production kinetics parameters are shown in Table 3 and fitted total gas production is presented in Figure 1. The ratio of cumulative gas production at 24 h and 48 h (GP24/GP48) were compared in an attempt to ascertain how much of the fermentation was completed in the first 24 h [7]. Similar to the ratio of 48 h cumulative gas production and asymptotic gas production, a (GP48/a) were compared in order to determine how close 48 h gas production was from a. As with chemical composition, values from the evaluated parameters varied between substrates. The asymptotic gas production values (a) ranged from 74.62 (wild asparagus) to 183.43 mL/g DM (Maltese bread), which was significantly higher than all other substrates. Loquat had the numerical highest fractional rate of fermentation (c) value, while prickly lettuce had the lowest (0.066 h^−1^). Regarding lag time (b), values varied from 2.32 h (loquat) to 4.17 h (wild asparagus). The final pH of substrates ranged between 6.8 and 6.9.

**Figure 2:**
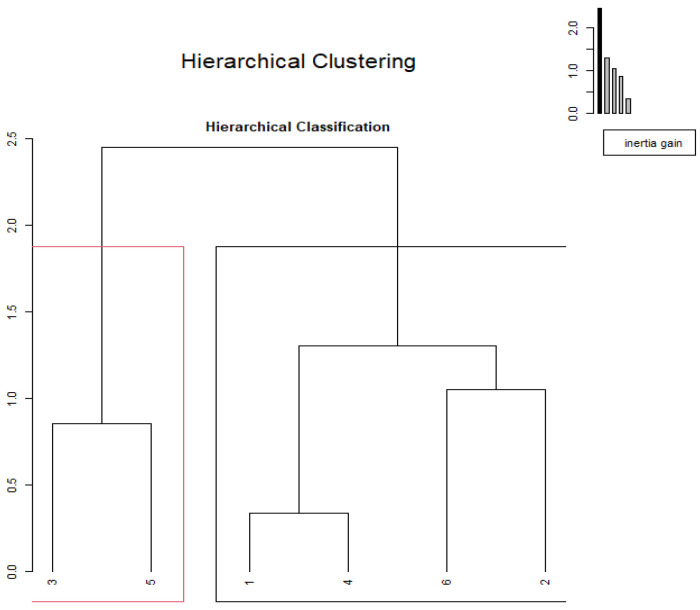
Hierarchical clustering of substrates using chemical composition.

**Figure 3:**
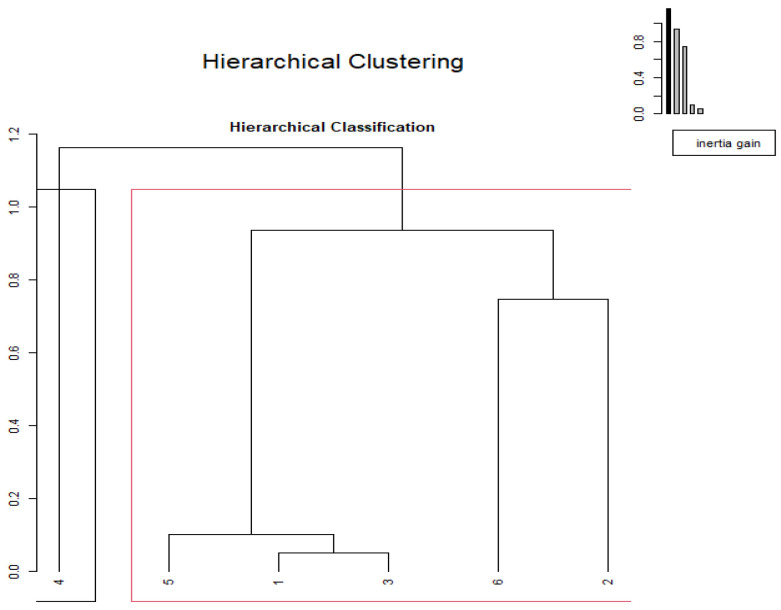
Hierarchical clustering of substrates using in vitro fermentation.

The cluster analysis using chemical composition data (Figure 2) showed a discrimination among substrates when data were used in a multivariate analysis. Cluster analysis detected the formation of two groups, the first being formed by wild asparagus and loquat (ashes and DM and EE) and the second being the rest of substrates, namely feed sheep, mature carob, bread, and prickly lettuce. A second analysis was performed on the fermentation kinetic data (Figure 3). Additionally, in this case, two clusters were identified; cluster A was singleton (wild asparagus) and B (others).

### 3.3. UV-Vis Analysis

#### Antioxidant Activity Assessment

**Figure 4:**
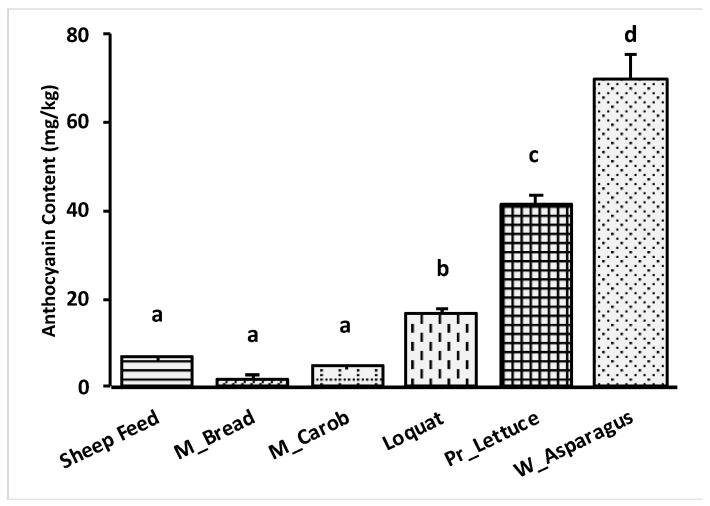
Anthocyanin content (mg/kg) of each feed sources. ^abcd^ Bars with different letters differ significantly at *p* < 0.05.

**Figure 5:**
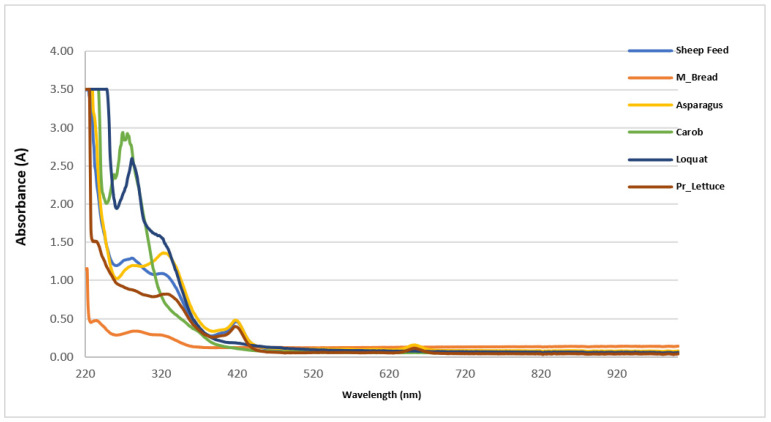
The UV-Vis profiles for each feed sources.

The results for the physicochemical parameters (color index, tint ratio, and flavonoid ratio) and anthocyanin content are illustrated in Table 4 and Figure 4, respectively. The color index ranged between 0.3 A and 26.5 A and was exhibited most strongly by wild asparagus and prickly lettuce, whereas Maltese bread had the lowest value (0.301 A). The tint ratio ranged between 6.52 and 0.99, respectively for prickly lettuce and Maltese bread. The flavonoid ratio ranged between 0.02 and 0.23, respectively, for mature carob and Maltese bread. The anthocyanin measured by the spectrophotometric method showed values that differed significantly among feed sources as shown in Figure 5. Maltese bread was followed by mature carob and sheep feed, respectively, 1.7 ± 0.83, 4.75 ± 0.28 and 6.63 ± 0.47 mg/kg. The highest values were detected in wild asparagus and prickly lettuce, followed by loquat, respectively, 69.8 ± 5.7, 41.2 ± 2.4, and 16.6 ± 1.28 mg/kg. In detail, Maltese bread, mature carob, and sheep feed showed similar anthocyanin content (average value of 4.36 mg/kg), resulting significantly lower than loquat, prickly lettuce, and wild asparagus that showed the highest values settled around an average of 42.53 mg/kg.

Figure 5 shows the UV-Vis profiles expressed as different wavelengths (nm) and relative absorbance (A) of each feed source. As shown in Table 5, the Maltese bread reported the highest values of red and blue flavonoids, respectively, 33 and 34.2%, while the highest values of yellow flavonoids were detected in prickly lettuce, sheep feed, and wild asparagus, respectively, 77.1, 75.4, and 71%. On the contrary, Maltese bread reported the lowest values of yellow flavonoids (32.8%), while the lowest values of red and blue flavonoids were detected in prickly lettuce (12 and 11%), sheep feed (13 and 12%), and wild asparagus (15.3 and 14%).

### 3.4. FC Test Results

**Figure 6:**
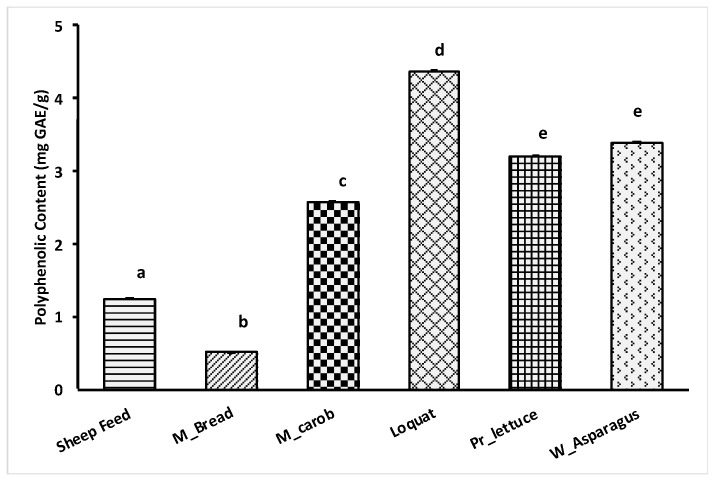
The polyphenolic content (mg GAE/g) of each feed source. ^abcde^ Bars with different letters differ significantly at *p* < 0.05.

The results for Folin–Ciocalteu assay are illustrated in Figure 6. The results are expressed as mg of Gallic acid equivalents (GAE). As shown, feed resources with the highest polyphenols content were loquat (4.4 mg GAE/g), which significantly differed from wild asparagus and prickly lettuce (3.3 mg GAE/g average value) and from mature carob (2.6 mg GAE/g) (*p* < 0.05). Sheep feed (1.26 mg GAE/g) and Maltese bread (0.52 mg GAE/g) were around an average of 0.9 mg GAE/g and were, hence, the lowest in average value.

### 3.5. DPPH Assay

**Figure 7:**
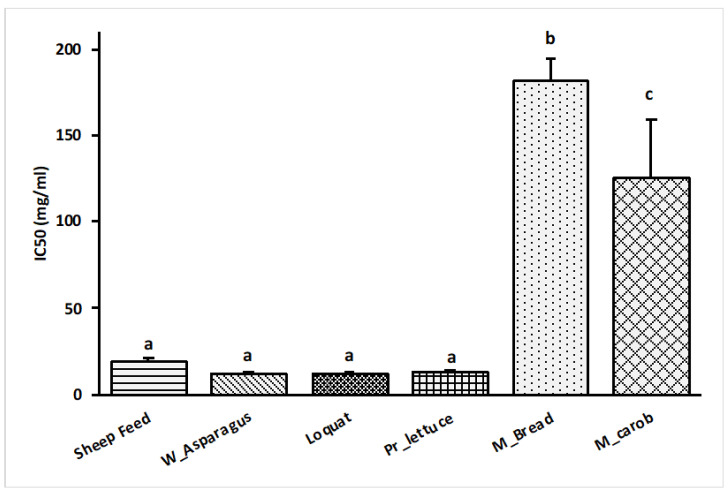
IC_50_ values (mg/mL) of each feed sources in relation to DPPH free radical scavenger. ^abc^ Bars with different letters differ significantly at *p* < 0.05.

The DPPH values are reported in Figure 7, showing the highest average values for Maltese bread (182 mg/mL) that differed significantly from mature carob (125.6 mg/mL) and the rest of the substrates. Loquat, wild asparagus, prickly lettuce, and sheep feed with the following content equal to 11.2, 11.4, 12.7, 19.2 mg/mL, respectively, indicated that they were more active than others (*p* < 0.05).

## 4. Discussion

In the present study, we analyzed non-conventional feed resources (NCFR), except for sheep feed, which are widely distributed in the Maltese Islands and for this reason, their potential use in animal feeding raised interest mostly in local producers. NCFR generally refer to all those feeds that were not traditionally used for feeding livestock and are not commercially used in the production of livestock feed. Defined in this manner, NCFR can be looked at as covering a wide diversity of feeds and their nutrient contents. The feasibility of using NCFR depends on their feed value as well as their characteristics: the nutritive value is determined by the concentrations of its chemical components, as well as their rate and extent of digestibility. Thus, the efficient use of NCFR relies on their chemical and physical properties, which influence production system outputs. Since the scarcity of knowledge related to the chemical composition and nutritive value limits their use in feeding animals, it was important to analyze their chemical composition and fermentation kinetics.

As a general observation, chemical composition varied largely between the different sources analyzed. Variation in chemical composition could possibly be influenced by several factors, such as the variety, cultivation, harvesting, storage, and food processing techniques [8,9,10,11].

Wild asparagus reported higher values for both NDF and ADF related to the cultivated varieties [12]. Its greater fibrousness could be related by the harvesting time and determined by the micro-environment in which each spear grew and, in particular, by the prevalence of direct or diffused solar radiation [13]. High levels of NDF and ADF were found also in mature carob, loquat, and prickly lettuce. However, there are no existing data related to the chemical composition of loquat and prickly lettuce.

Concerning the prickly lettuce (*Lactuca serriola* L.), the main efforts were focused on morphological and, more recently, genetic characterization since the wild varieties represent potential genetic resources for improving nutritional traits in modern crop types [14]. For this reason, the metabolic aspects were barely taken into account, especially for the wild type. No data related to the content of NDF and ADF were found. However, crude protein, ether extract, ash content, and moisture content were in accordance with those reported by other authors [15].

Few studies observed differences in the aspects of chemical characterization of *Eriobotrya japonica* fruit and its parts (skin, pulp, seed, and starch) such as the vitamins, minerals, moisture, and proteins [16,17,18]. The chemical composition of the leaves present in our study is in accordance with the literature, except for ash content, which was higher than those reported by Hernández [19]. Ash is a reflection of the amount of mineral elements in the samples and, therefore, serves as the main source of mineral elements needed for human and animal health. Loquat leaves have a high amount of minerals, especially potassium, calcium, magnesium, iron, and sodium [20]. We speculated that the high content found in this study may depend on soil characteristics. The quantity and quality of ash depends on a large amount of factors including plant type, plant fraction, growing conditions, fertilization, choice of harvest date, harvest techniques, and conversion systems [21]. Carob (*Ceratonia siliqua* L.) is widely used as a source of fiber in animal feed in the Mediterranean region. The chemical composition of carob pods can vary with genetic, environmental, climatic factors, geographical origin, and harvesting season. The plant type and cultivar significantly influence chemical composition and biological activities of the carob pod [22]. Generally, it is characterized by high content of dietary fiber, hence classifying it as fibrous resource. Despite the NDF content being higher than those reported by Richane [23], NDF values of the studied carob samples were within the recommended range of 17–33 g/100 g DM for ruminants [23]. In general, carob is low in protein (3–4%) and lipid content (0.4–0.8%) [24]. The crude protein content in our study was above the minimum level required for maintenance of ruminants (6–8 g/100 g DM) [23] and was higher than those reported by Calislar and Kaplan [25] (4%) but similar to the one reported by Youssef [26] (6.3%). Nowadays, the main application of the pods is as animal feed. In fact, despite the limited information available concerning the chemical composition, nutritive values, and ruminal digestion of carob pods, it could represent a potential and economic alternative for grazing animals [27].

As expected, the wide range detected in chemical composition resulted in variability in rumen fermentation kinetics. In general, the fermentation parameters of feed varied according to the ingredients (*p* = 0.007). Additionally, there is little information on bread and no data related on the Maltese bread. Some evidence suggests that the chemical composition of the Maltese bread may be within the expected nutritional values [28]. An alternative feedstuff in feeding livestock is the bread by-product (BBP). Among the food industry by-products, bread waste from the bakery is an important source. The major restriction of feeding BBPs to livestock is the variation in the chemical composition [29]. However, some evidence suggested that diets including high levels of BBPs (30–45%) were shown to be better in vitro rumen degradation of starch, while showing a lower degradation of crude protein and fiber [30]. Maltese bread showed the highest volumes of gas production (a) and one of the highest rates of fermentation (c). These results indicated that Maltese bread fermented more extensively and at a faster rate than most of the other feed resources used in this study. Higher gas production indicates higher fermentative activity and higher nutrient degradation by rumen microorganisms. In fact, the carbohydrates’ fractions differently affect the fermentation kinetics: starch promotes a more intense and rapid process, while conversely, structural carbohydrates cause a slower and less consistent fermentation, limiting the access to the cell content by micro-organisms, reducing nutrients’ degradability, and slowing down the fermentation rate. The ratio between GP-24 h and GP-48 h was higher in Maltese bread than other substrates implying that a greater extent of fermentation had taken midway through the incubation period, showing a higher fermentation efficiency. This seemed to be confirmed by the fact that loquat, prickly lettuce, and especially wild asparagus showed the lowest volumes of gas production, indicating a lower fermentation kinetics. All these resources had a high content of NDF and ADF. The negative correlation between NDF level and gas production was consistent with other studies [31,32], in which a reduction in gas production with increasing of NDF content of the substrates was reported. In general, the present results were lower than those found in the literature [33,34]. Aderinboye et al. [33] found that cumulative gas production and the rate of fermentation were generally higher with cattle than sheep ruminal fluid. The same result was consistent with Cone et al. [34]; however, the activity of the rumen fluid is not only determined by the donor animal, but also by the ration of the animal.

As was highlighted by most authors and reviews [35], the composition and type of diet given to the donor animals determined the fermentative capacity and the degree of the adaptation of the analyzed substrates. Thus, the rate and extent of fermentation of a fibrous or a concentrate feed highly depended on its evaluation with inoculum from an animal feed on either a forage- or a concentrate-type diet [36]. Therefore, it is recommended to feed donor animals with a diet similar to the substrate to be incubated in vitro, or to the in vivo feeding conditions that are intended to be studied. In fact, in the present paper, these conditions were met. Rather, we hypothesized that such a result can also be due to the storage (frozen inocula) that could have affected the microbial activity declining over time and influencing the fermentation kinetics of the substrates [37,38]. Lag time is indicative of the time taken for microbes to adhere themselves to the substrates, and microbial attachment to insoluble substrate. In the present study, no significant difference was found in c parameter to underline a homogeneous pre-condition for digestion to proceed. However, the main advantage of using frozen inocula remains justified, since the use of fistulated animals is avoided, aliquots can be prepared and used for more than one fermentation and results are repeatable as highlighted in our study. Moreover, results of gas production obtained from different researchers in vitro are often, in any case, difficult to compare because of the influence of several confounding sources of variation: the procedures of rumen fluid collection and treatment [39], the type of buffer used [40], the ratios among feed sample size, fermentation fluid, headspace volume [41], and the type of GP equipment, such as syringes or bottles that can be closed or vented at fixed times or at fixed pressure [42,43,44]. Considering sheep feed as a control since it is a reference feed in sheep nutrition, with regard to the cumulative gas production, we observed that other feeds, except for wild asparagus, behave in a similar way, suggesting a substrate interchangeability.

In the present study, all the feed resources were evaluated for their content of bioactive compounds as phenolic contents and for their antioxidant activity. The exogenous antioxidants were mainly derived from food and medicinal plants, such as fruits, vegetables, cereals, mushrooms, beverages, flowers, spices, and traditional medicinal herbs [45,46]. It was found that phenolic compounds are amongst the most effective antioxidant constituents in plant foods, including fruits, vegetables, and grains [47]. They are a ubiquitous group of phytochemicals that possess different physiological activities associated with their chemical structures. Considering their important health effects, the efficient extraction methods of natural antioxidants, appropriate assessment of antioxidant activity as well as their main resources from food and medicinal plants drew great attention in food science and nutrition.

Although carob is widely used as a source of fiber in animal feed, it holds potentially significant importance due to its phytochemical constituents with functional properties, flavoring properties, and nutrition benefits [48]. In the present study, the polyphenol content of mature carob was in accordance with the literature, while the anthocyanin content showed lower values [23]. The anthocyanin content may be correlated to irradiance levels at which the plants were grown [49]. The antioxidant activity was lower than what was reported in the literature. In fact, it was demonstrated that the antioxidant activity decreased significantly throughout the ripening process [50]. For this reason, the maturity of the carob may have influenced the antioxidant power of this phytochemical group.

Several studies reported different bioactive compounds, associated with loquat health-relevant properties, making its use in food industry desirable. Bioactivity was observed in various parts of the plant and its fruit, including the leaves [51]. In leaves, the antioxidant capacity is linked to the phenolic compounds [52]. Some authors [46,53,54,55] demonstrated a linear correlation between the content of total phenolic compounds and their antioxidant capacity. Our results showed a similar polyphenolic content compared with that reported by other authors [56]. The low IC_50_ value found herein and flavonoid content indicated a valid antioxidant activity in loquat leaves.

Many studies investigated the diversity and abundance of polyphenols in different types and cultivars of asparagus under various growing conditions [57,58]. The content of polyphenols in our study was similar to that reported by di Maro [59], but lower than those reported by other authors [60,61]. The content of anthocyanins was lower than those reported by Dong et al. [62]. The DPPH scavenging activity was similar to that reported by Adouni et al. [61], but lower than that reported in fresh spears [59].

Although several plants of the *Lactuca* genus were examined for their chemical constituents, prickly lettuce was not investigated in detail for its phytochemical constitution. We reported a high content of anthocyanins (41.2 mg/mL), compared to the literature [63,64]. The DPPH scavenging activity was in accordance with the literature [65], indicating a valid antioxidant activity in prickly lettuce. In general, mature carob, wild asparagus, prickly lettuce, and loquat could be potentially used as a source of fiber and with reference to the last three feeds, our data suggested that the highest DPPH radical scavenging activity make them also as valid antioxidant feeds.

Additionally, there were no data related on the Maltese bread. The antioxidant activity of bread is usually attributed to the presence of Maillard reaction products that are known to possess free-radical scavenging activities [66]. In this case, lower antioxidant activity was noted for the Maltese bread in comparison to the other substrates. This could be correlated with the baking process. In fact, during bread baking, various modifications in the chemical composition and properties of the food matrix take place, leading to changes in the nutritional value of the final product [67]. This treatment can lead to the production of newly formed compounds responsible for different biological activities [68].

Finally, the antioxidant power of sheep feed was also evaluated. The lower IC_50_ value found herein indicated a valid antioxidant activity in sheep feed. This could be correlated with the presence of different type of wheat (semolina, bran, corn), and alfalfa which are rich sources of antioxidant flavonoids and phenolic compounds reported to have anti-inflammatory and antioxidant activity [69,70]. Additionally, it was previously reported that different varieties of sugarcane show good antioxidant properties [71]. In fact, the antioxidant activity is susceptible to variation among varieties, growing practices, processing and storage conditions on the biologically active compounds.

Principal component analysis was conducted on all six feeds to determine any latent differences between them, taking into account all the physicochemical characteristics under study. Spearman correlation (Table 5) revealed that there were strong positive correlations between color intensity, tint, % yellow, anthocyanin content, ash, ADF, and polyphenols (r_s_ = 0.714–1.000), indicating that the physical parameters correlate with the chemical constitution of these feeds. Additionally, anthocyanins and polyphenols exhibited a positive correlation (r_s_ = 0.771) but a negative correlation between anthocyanins and DPPH (r_s_ = −0.829), indicating that anthocyanins contributed to the total polyphenolic content whilst also contributing to the antioxidant activity of the feeds.

**Figure 8:**
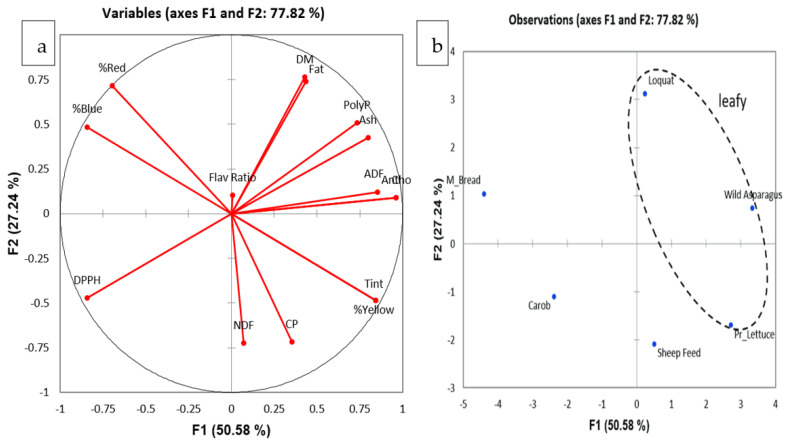
Principal component analysis (PCA) analysis of the six feed sources for the physicochemical, proximate and antioxidant parameters; (**a**) the factor loading plot demonstrating the different groups of variables; (**b**) the factor scores of the two latent factors.

Additionally, DPPH correlated negatively with most parameters (r_s_ < −0.486), indicating that most feed components (fiber, fat, and polyphenols amongst others) contribute to the antioxidant activity of these natural sources. Two latent factors had an eigenvalue greater than 1, which together explained 77.82% of the total variance (Figure 8a). The factor loadings demonstrated the different groups of variables. The first factor was loaded mostly on physical parameters, polyphenolic content, and antioxidant activity, while the second factor was loaded on most proximate parameters. Figure 8b demonstrates the factor scores of the two latent factors. Factor 1, on the horizontal axis, demonstrates the clustering of leafy feed sources (loquat, wild asparagus, and prickly lettuce) on the right-hand side of the scatter plot, with the rest more shifted towards the right. Factor 2, on the vertical axis, demonstrates the clustering of feeds, at the bottom of the plot, with low content of dry matter and fat, whilst a high content of protein and fiber. These demonstrate that although the leafy feed sources are distinctive from the rest, they may share some common properties with other feeds, indicating that all feed sources may provide benefit to ruminants in one way or another.

## 5. Conclusions

This study provided a chemical, antioxidant, and kinetics evaluation of different feed resource, hence giving a first insight in the characterization of some local feeds such as wild asparagus, pricky lettuce, and loquat. The results of this first investigation suggest the possibility of using local feeds in small ruminant nutrition with the advantage that, being local natural resources, they are better adapted to the climate and agronomic conditions and limit the environmental impact. Moreover, further research will be needed in order to establish the right amounts of the feedstuffs to be added in the diets of livestock based on animal production responses and, hence, their economic value.

## Figures and Tables

**Table 1:** Part of the feed resources tested.

Feed Resources	Scientific Name	Part Tested
Wild asparagus	*Asparagus aphyllus*	Aerial parts
Prickly lettuce	*Lactuca serriola*	Leaves
Loquat	*Eriobotrya japonica*	Leaves
Mature carob	*Ceratonia siliqua*	Pods
Maltese bread	Ħobża Maltija *	Crust and crumb

* Common name.

**Table 2:** Chemical composition (%) of individual feedstuffs. DM, dry matter; CP, crude protein; EE, ether extract; NDF, neutral detergent fiber; ADF, acid detergent fiber; ash content.

Feed Sources	DM	CP	EE	NDF	ADF	Ash
Sheep Feed	89.50 ± 0.04	14.2 ± 0.40	3.57 ± 0.03	46.64 ± 0.30	19.70 ± 0.40	9.70 ± 0.20
M_bread	90.4 ± 0.11	6.60 ± 0.15	2.90 ± 0.01	39.92 ± 0.42	4.36 ± 0.25	7.31 ± 0.17
W_asparagus	93.47 ± 0.14	8.90 ± 0.16	5.24 ± 0.18	50.45 ± 1.56	46.38 ± 0.53	12 ± 0.30
Pr_lettuce	90.05 ± 0.08	12.31 ± 0.18	2.31 ± 0.05	52.92 ± 0.38	38.64 ± 0.34	12.24 ± 0.27
Loquat	91.62 ± 0.09	5.70 ± 0.30	6.43 ± 0.05	36.80 ± 0.91	26.77 ± 0.58	17.08 ± 0.25
M_carob	87.75 ± 0	6.33 ± 0.07	0.01 ± 0	56.22 ± 0.32	25.80 ± 0.45	5.27 ± 0.03

**Table 3:** a = asymptotic gas production (mL/g DM incubated); b = lag time (h); c = fractional rate of fermentation (h^−1^); SEM = standard error of the mean; ns = not significant. ^a,b,c^ within column, means with different superscripts indicate statistical significance.

Substrate (S)	a	b	c	a24	a 48	24/48	48/a
Sheep feed	134.20 ^bc^	2.87	0.080	85.41 ^b^	121.25 ^ab^	0.663 ^b^	0.88 ^ab^
Loquat	113.96 ^ab^	2.32	0.210	71.098 ^b^	99.75 ^ab^	0.675 ^b^	0.85 ^ab^
M_carob	152.00 ^bc^	2.48	0.078	100.47 ^ab^	142.54 ^a^	0.706 ^ab^	1.00 ^ab^
W_asparagus	74.62 ^a^	4.17	0.080	45.86 ^c^	66.32 ^b^	0.691 ^b^	0.88 ^ab^
Pr_lettuce	111.29 ^ab^	2.64	0.066	67.31 ^b^	96.02 ^ab^	0.700 ^ab^	0.86 ^ab^
M_bread	183.43 ^c^	3.64	0.159	170.26 ^a^	188.08 ^ab^	0.960 ^a^	1.01 ^a^
SEM	8.55	0.26	0.03	9.87	10.5	0.002	0.03
P	0.007	ns	ns	<0.001	<0.001	0.005	0.016

**Table 4:** The color index (Abs), Tint ratio (A420/A520), and Flavonoid ratio (A520/A280) for each feed sources.

Feed Sources	Color Index (Abs)	Tint Ratio	Flavonoid Ratio
Sheep feed	3.02 ± 0.15	5.91 ± 0.23	0.05 ± 0.002
M_bread	0.3 ± 0.15	0.99 ± 0.013	0.23 ± 0.08
W_asparagus	26.5 ± 1.06	4.67 ± 0.39	0.09 ± 0.009
Pr_lettuce	20.2 ± 0.49	6.52 ± 0.44	0.07 ± 0.004
Loquat	3.55 ± 0.24	1.88 ± 0.041	0.03 ± 0.001
M_carob	1.04 ± 0.05	1.85 ± 0.045	0.02 ± 0.008

**Table 5:** Spearman correlation matrix for the physicochemical parameters of the feed sources.

Variables	Tint	%Red	%Yellow	%Blue	Antho	Flav Ratio	DM	Ash	CP	Fat	NDF	ADF	PolyP	DPPH
CI	0.714	−0.600	0.714	−0.714	1.000	0.086	0.543	0.714	0.257	0.429	0.143	0.943	0.771	−0.829
Tint		−0.943	1.000	−1.000	0.714	−0.029	−0.029	0.543	0.714	0.086	0.257	0.543	0.314	−0.486
%Red			−0.943	0.943	−0.600	0.086	0.257	−0.257	−0.771	0.200	−0.543	−0.486	−0.143	0.257
%Yellow				−1.000	0.714	−0.029	−0.029	0.543	0.714	0.086	0.257	0.543	0.314	−0.486
%Blue					−0.714	0.029	0.029	−0.543	−0.714	−0.086	−0.257	−0.543	−0.314	0.486
Antho						0.086	0.543	0.714	0.257	0.429	0.143	0.943	0.771	−0.829
Flav Ratio							0.543	0.029	0.371	0.143	−0.257	−0.029	−0.314	0.200
DM								0.600	−0.200	0.771	−0.543	0.486	0.543	−0.600
Ash									−0.029	0.657	−0.429	0.600	0.771	−0.886
CP										−0.143	0.257	0.029	−0.371	0.143
Fat											−0.771	0.257	0.543	−0.714
NDF												0.314	−0.086	0.257
ADF													0.829	−0.771
PolyP														−0.943

## Data Availability

The data presented in the current study are available from the corresponding authors on reasonable request due to privacy or ethical restrictions.
